# From patient classification to optimized treatment in ART: the AMPLITUDE Delphi consensus

**DOI:** 10.3389/frph.2024.1467322

**Published:** 2024-09-27

**Authors:** Christophe Blockeel, Anne Guivarc’h-Leveque, Catherine Rongieres, Nelly Swierkowski-Blanchard, Géraldine Porcu-Buisson, Chadi Yazbeck, Christine Wyns

**Affiliations:** ^1^ Brussels IVF, Centre for Reproductive Medicine, Universitair Ziekenhuis Brussel, Vrije Universiteit Brussel, Brussels, Belgium; ^2^ Service of Reproductive Medicine, Clinique Mutualiste La Sagesse, Rennes, France; ^3^ Department of Reproductive Medicine, Strasbourg University Hospital, Strasbourg, France; ^4^ Reproductive Medicine Center, Intercommunal Hospital Center, Poissy, France; ^5^ RHuMA-TEAM, UMR-BREED, UFR-SVS, UVSQ, Montigny-Le-Bretonneux, France; ^6^ Department of Reproductive Medicine, Institut de Médecine de la Reproduction, Marseille, France; ^7^ Obstetrics Gynecology and Reproductive Medicine, Reprogynes Medical Institute, Paris, France; ^8^ Obstetrics Gynecology and Reproductive Medicine, Groupe Hospitalier Privé Ambroise Paré Hartmann, Neuilly-sur-Seine, France; ^9^ Department of Gynecology-Andrology, Cliniques Universitaires Saint-Luc, Brussels, Belgium

**Keywords:** ovarian stimulation, assisted reproductive technology, gonadotropin, dose personalization, ovarian response

## Abstract

**Introduction:**

A Delphi consensus was performed to evaluate expert opinions on the management of key aspects of ovarian stimulation.

**Methods:**

A Scientific Committee developed eleven statements for patient profiles corresponding to predicted ovarian responses (low, normal, and high) based on antral follicle count (AFC) and anti-Müllerian hormone (AMH). The statements were distributed (online survey) to French and Belgian fertility specialists. Consensus was reached when ≥66.7% of participants agreed or disagreed.

**Results:**

Among 52 respondents, a consensus agreement was reached for each patient profile for personalizing the initial dose of gonadotropin, taking age, weight, body mass index, nature of the cycle, and the decision to perform a fresh transfer or a freeze-all strategy into consideration. The respondents preferred a fresh transfer for low and normal responders and a freeze-all strategy in case of high risk of hyperstimulation, newly diagnosed uterine or tubal pathology and premature progesterone elevation. A consensus was reached for 10–15 oocytes as optimal oocyte target from the first round of voting. The panel agreed to increase the gonadotropin dose in case of insufficient response and preferred a GnRH antagonist protocol for a subsequent cycle in case of excessive response. Finally, a consensual answer was obtained for using LH/hCG activity in case of hypogonadotropic hypogonadism, advanced age, inadequate response during first stimulation and suspected FSH receptor polymorphism.

**Discussion:**

The AMPLITUDE consensus supports the importance of optimizing the ovarian stimulation protocol for patients undergoing assisted reproductive technology treatment. Additional studies could complete these findings and guide fertility specialists in their daily practice to improve ovarian stimulation outcomes.

## 1 Introduction

In recent decades, infertility has become a public health problem, with one out of six couples experiencing the absence of natural conception over a 1-year period (1). In 2019, more than 1 million treatment cycles, across 40 European countries were reported to the European IVF-monitoring (2). Infertility treatment success depends largely on a successful ovarian stimulation with increasing live birth rates after a fresh transfer obtained for up to 15 oocytes, reaching a plateau between 15 and 20 oocytes before decreasing, based on a large real-world cohort (3). Some complications can occur during fertility treatment, with the most reported complication being ovarian hyperstimulation syndrome (OHSS) (2). Thus, several methods have been recently discussed to limit the incidence and severity of OHSS, among them the personalization of the starting dose (4).

Since the early years of assisted reproductive technology (ART), patients have been classified as “low, good, or high” responders at the first attempt of treatment. Although these terms are widely used in clinical practice, there is still no consensus as to their exact definitions. Indeed, despite its importance to ART, the methodology for classifying high responder patients remains heterogeneous (5). Although the Bologna criteria have proven useful in identifying patients with low response, these criteria have been challenged because they carry the risk of grouping together women with very different biological characteristics, whose treatment calls for different strategies. Subsequently, the POSEIDON classification proposed a different classification including, besides ovarian reserve markers, two age categories and oocyte output at the previous attempt (6).

In routine practice, the classification of treatment-naive patients is based on the assessment of the ovarian reserve. The combined use of an ultrasound marker, antral follicle count (AFC) and a biochemical marker, anti-Müllerian hormone (AMH), is considered as a good predictor of ovarian response (7, 8). Other parameters such as age, weight, menstrual cycle length, basal follicle stimulating hormone (FSH), and estradiol (E2)/FSH at the beginning of a cycle, can be used to establish the stimulation protocol and doses.

However, limited data are provided to clinicians on the management of the various aspects of ovarian stimulation. In 2020, the European Society of Human Reproduction and Embryology (ESHRE) working group on ovarian stimulation therefore drew up guidelines based on the data available in the literature. The level of evidence was very often limited and of low quality: of the 61 recommendations developed, nine out of ten were backed by low or very low-quality evidence (8).

In this context, the aim of this study was to provide an experts’ consensus of best practices regarding personalization of the gonadotropin dose by classifying patients undergoing ART in three distinct ovarian response profiles.

## 2 Material(s) and methods

### 2.1 Group definition based on expected ovarian response and statements development

In this survey, we used three patient profiles classically used in clinical practice. The expected ovarian response profile was based on the evaluation of basal AMH and AFC, the most robust parameters for assessing ovarian response according to literature (7, 9). Thus, the low response profile includes patients with impaired ovarian reserve, while the normal response profile is represented by patients with normal ovarian reserve, and the high response profile by patients at risk of ovarian hyperstimulation. To establish the statements included in the survey, topics important for treatment personalization in ART were selected based on data published in the literature and the ESHRE guidelines (Table 1).

**Table 1:** Rationale for DELPHI questionnaire statements.

Context	References used
Patient profile and personalization of the initial gonadotropin dose Currently, ovarian stimulation faces a significant challenge: improving the low success rate (birth rate achieved in a minimum number of attempts) [1]. Personalizing ovarian stimulation protocols could be one potential solution. However, as of now, no ovarian reserve test has proven to be comprehensive, in terms of both sensitivity and precision [2]. Furthermore, recent recommendations published by ESHRE have been noted as incomplete [3]. The objective of this section is to identify parameters enabling the customization of the initial treatment dose	1. Malinowski et al. (2016) 2. Barrenetxea et al. (2019) 3. Bosch et al. (2020)
Fresh transfer vs. freeze-all Initially implemented in cases of high risk of ovarian hyperstimulation syndrome, the use of freeze-all has since been expanded to other indications as a “planned strategy” aimed at improving implantation rates [4]. Therefore, the objective here is to identify the conditions for the use of freeze-all	4. Bourdon et al. 2020
Ovarian response There is limited data available to establish the follicle size most likely to produce a mature oocyte [1]. Authors set different thresholds, ranging from 12 mm to 19 mm [1, 2]. It has also been demonstrated that the live birth rate after a fresh embryo transfer cycle reaches a plateau with 15–20 oocytes retrieved [3]. Thus, live birth rates are comparably high when retrieving 4–9, 10–15, or >15 oocytes [4]. The aim of this DELPHI study is to clarify the experts’ stance on these triggering criteria and oocyte target, as well as the approach to take in cases of inadequate or excessive response	1. Abbara et al. (2018) 2. Papanikolaou et al. (2006) 3. Sunkara et al. (2011) 4. Drakopoulos et al. (2015)
LH/hCG activity The importance of LH/hCG activity during follicular growth is well established. However, its use during ovarian stimulation as part of an ART (Assisted Reproductive Technology) pathway remains controversial. While some authors have concluded that there is insufficient evidence to recommend LH/hCG supplementation, other studies demonstrate a positive effect of this supplementation on pregnancy rates [1]. The aim of this DELPHI study is to determine the conditions under which the addition of LH/hCG activity should be recommended	1. Lahoud et al., (2017)

### 2.2 Consensus participants

The AMPLITUDE program started by the set-up of the Scientific Committee, comprising seven ART specialists, leaders in Reproductive Medicine working in French and Belgian *in vitro* fertilization (IVF) centers. Particular care was taken to ensure that these experts came from different regions of France (Bretagne, Grand-Est, Ile-de-France, Provence-Alpes-Côte d'Azur) and Belgium (Brussels area). Each member of the Scientific Committee suggested about ten experts to respond to the questionnaire. In total, 71 experts were preidentified. The Scientific Committee ensured that this panel was representative for the geographical distribution of fertility specialists practicing in France and Belgium (Supplementary Table 1).

### 2.3 Consensus process

The AMPLITUDE consensus followed the Delphi methodology and was composed by three steps. The first step consisted of the development of the statements by the seven Scientific Committee members. This step took place during two virtual meetings. After these meetings, definitive statements were approved by the Scientific Committee. In the second step (from December 06, 2022 to January 11, 2023) the questionnaire was disseminated in French and English languages via an online survey for the first round of voting, to the preidentified panel of experts. Eleven statements were provided, and each participant expressed anonymously their level of agreement on a four-point Likert scale, from fully agree (1) to fully disagree (4). Consensus was considered to be achieved if the proportion of participants either agreeing with a statement (responding “somewhat agree” or “fully agree”) or disagreeing with a statement (responding “somewhat disagree” or “fully disagree”) was at least 66.7%. The third step began after the analysis of the responses and its communication to the Scientific Committee during a virtual meeting held in January 2023. Statements that did not achieve consensus in the first round were discussed and if needed, revised by the Scientific Committee. The newly reworded statements as well as the results of the first round were shared with the participating experts for the second round of voting, from March 15, 2023 to May 15, 2023. The final analyses were performed and presented to the Scientific Committee on June, 2023. No new statements were proposed as such addition is inconsistent with the principles of the Delphi method.

This Delphi consensus was coordinated by KPL Agency, a Medical Education company, who handled contacts and meetings with the Scientific Committee, distribution of the questionnaire and analysis of the results.

## 3 Results

### 3.1 Statements

The 11 statements approved by the Scientific Committee that were voted on in the first and second round are shown in Supplementary Table 2. These statements are related to the personalization of the initial gonadotropin dose (3 statements), fresh transfer *vs*. freeze-all (2 statements), ovarian response (5 statements), and LH/hCG activity (1 statement).

The results obtained accurately reflect the collective opinion of the experts at a specific point in time, in accordance with the methodological criteria established from the outset.

### 3.2 Participants

Overall, 52 of the 71 preidentified experts completed the entire survey during the first round (73% response rate). Among them, 41 were based in France (79%) and 11 in Belgium (21%). These 52 IVF specialists were contacted again to take part in the second round, and 51 of them responded to the questionnaire (98% response rate). As shown in Supplementary Table 1, regional distribution is representative of the geographical spread of fertility specialists in the two countries.

### 3.3 Patient profile and personalization of the initial gonadotropin dose

*Statement 1. The initial* *dose of gonadotropins administered should be personalized for each patient profile: low, normal or high response profile.*

**Figure 1:**
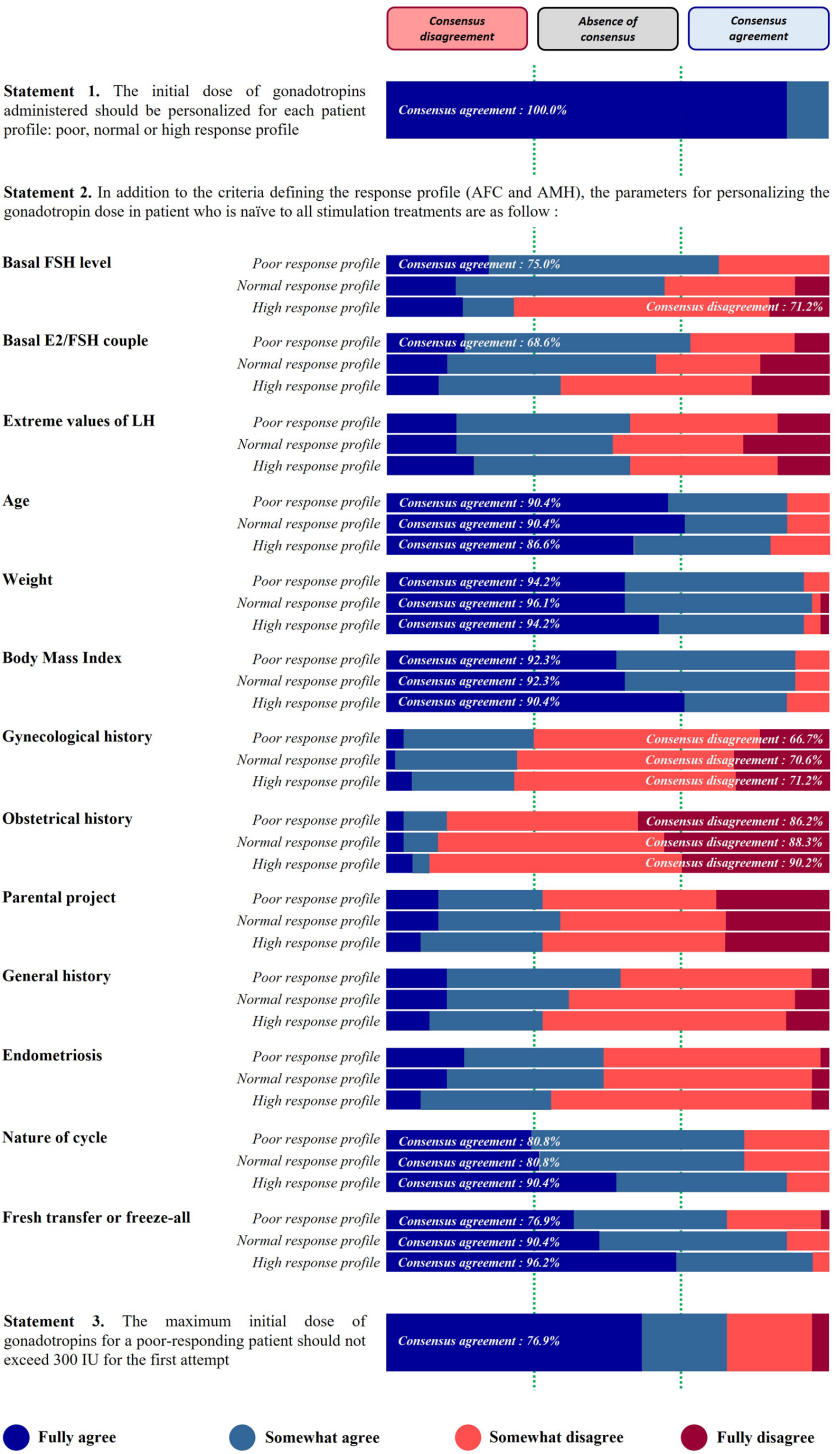
Patient profile and personalization of the initial gonadotropin dose.

This statement received 100% of agreement from the panel of experts and 90.4% of the respondents fully agreed (Figure 1).

*Statement 2. In addition to the criteria defining the response profile (AFC and AMH), the parameters for personalizing the gonadotropin dose in a patient who is naïve to all stimulation treatments are…*

The relevance of using hormone levels, anthropometric measurements, medical history, and patient objectives in order to personalize the dose of gonadotropins is shown in Figure 1.

A high proportion of respondents agreed to take into account the age, weight, and body mass index (BMI) to personalize the dose, regardless of the patient profile. For these three anthropometric parameters, the agreement consensus was very high (90% of respondents or more).

Among the hormones listed, a consensus agreement was reached for the use of basal FSH level and basal E2/FSH couple in the low response profile only (75.0% and 68.6%, respectively). On the other hand, for the high-response profile, 71.2% of the panel of specialists disagreed to use the basal FSH level. No consensus was reached for extreme values of LH (whatever the response profile), basal E2/FSH ratio (in normal and high response profiles), and basal FSH level (in normal response profile).

A strong level of agreement was also reached for the following two proposals: nature of the cycle (ovulatory, dysovulatory, anovulatory) (80.8% for low and normal response profiles and 90.4% for high response profile) and the decision to perform a fresh transfer or a freeze-all (76.9%, 90.4%, 90.6%, for low, normal, and high response profile, respectively). In contrast, gynecological and obstetrical history were not considered relevant by the respondents. The disagreement consensus was strong for obstetrical history (history of uterine rupture, ectopic pregnancy…), approaching or exceeding 90% of disagreement for the three profiles. The results are more nuanced for gynecological history (pelvic surgery), where the disagreement consensus threshold is barely exceeded for the three profiles (66.7%, 70.6%, and 71.2% for low, normal, and high responders, respectively). No consensus was reached on the last proposals, i.e., parental project (desired number of children), general history (chronic inflammation disease, cancer, *etc*.), and endometriosis (stage and phenotype).

*Statement 3. The maximum initial dose of gonadotropins for a low-responding patient should not exceed 300 IU for the first attempt.*

This statement received a 76.9% agreement from the panel of experts (Figure 1).

### 3.4 Fresh transfer vs. freeze-all

*Statement 4. When initiating ovarian stimulation, a fresh transfer strategy is preferred as the first-line treatment.*

**Figure 2:**
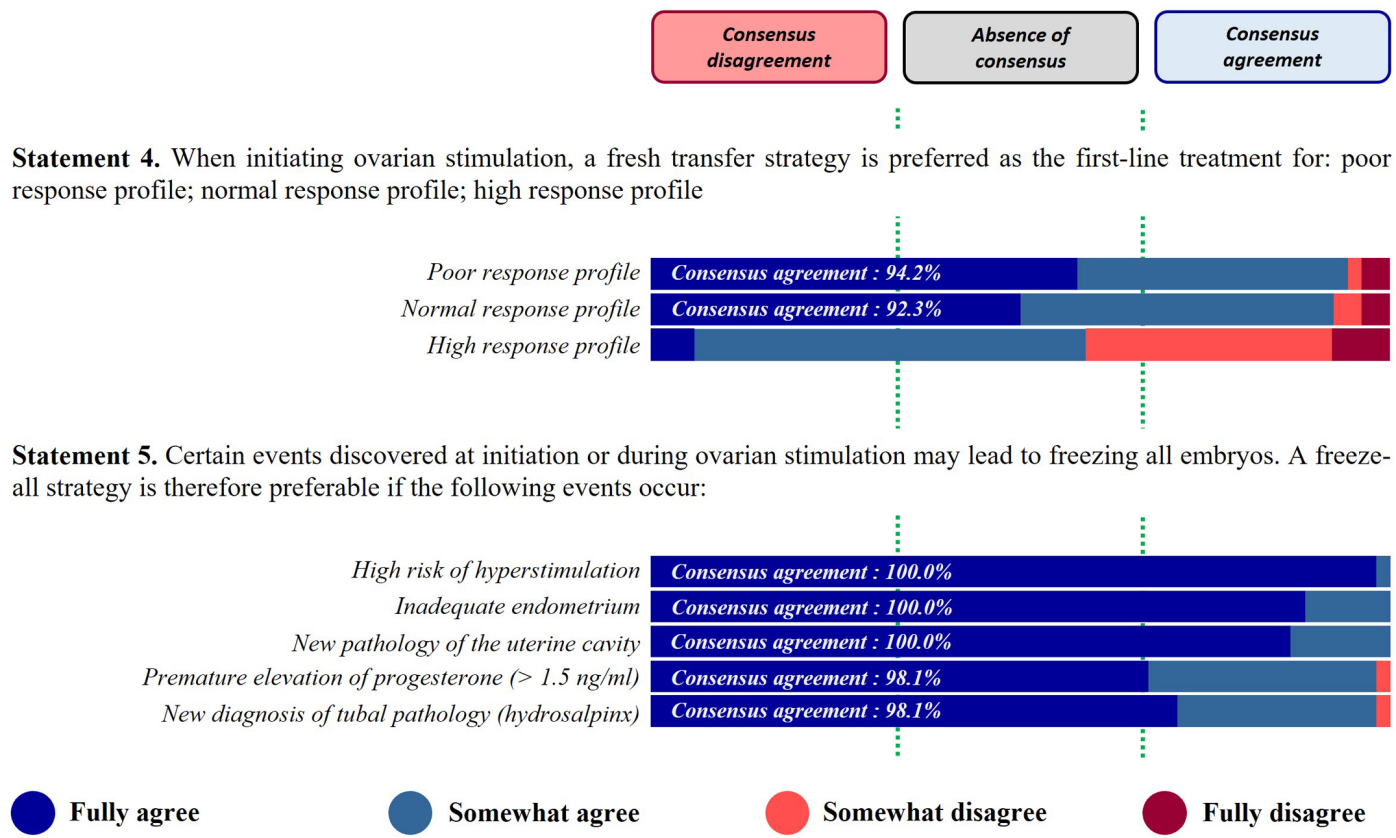
Fresh transfer vs. freeze-all.

A strong consensus agreement was reached for low (94.2%) and normal (92.3%) responders, suggesting that a fresh transfer strategy should be preferred to a freeze-all strategy, at the start of the ovarian stimulation. In contrast, no consensus was obtained for high responders, for this statement (Figure 2).

*Statement 5. Certain events discovered at initiation or during ovarian stimulation may lead to freezing all embryos. A freeze-all strategy is therefore preferable if the following events occur…*

This statement received a strong consensus agreement for the different proposals listed, with a total agreement for three of them: high risk of hyperstimulation (100%); inadequate endometrium (100%); new pathology of uterine cavity (100%); premature elevation of progesterone >1.5 ng/ml (98.1%); new diagnosis of tubal pathology (hydrosalpinx) (98.1%) (Figure 2).

### 3.5 Ovarian response

*Statement 6. During ovarian stimulation with an antagonist protocol in fresh transfer, a number of 3 follicles measuring ≥17 mm is a trigger criterion.*

**Figure 3:**
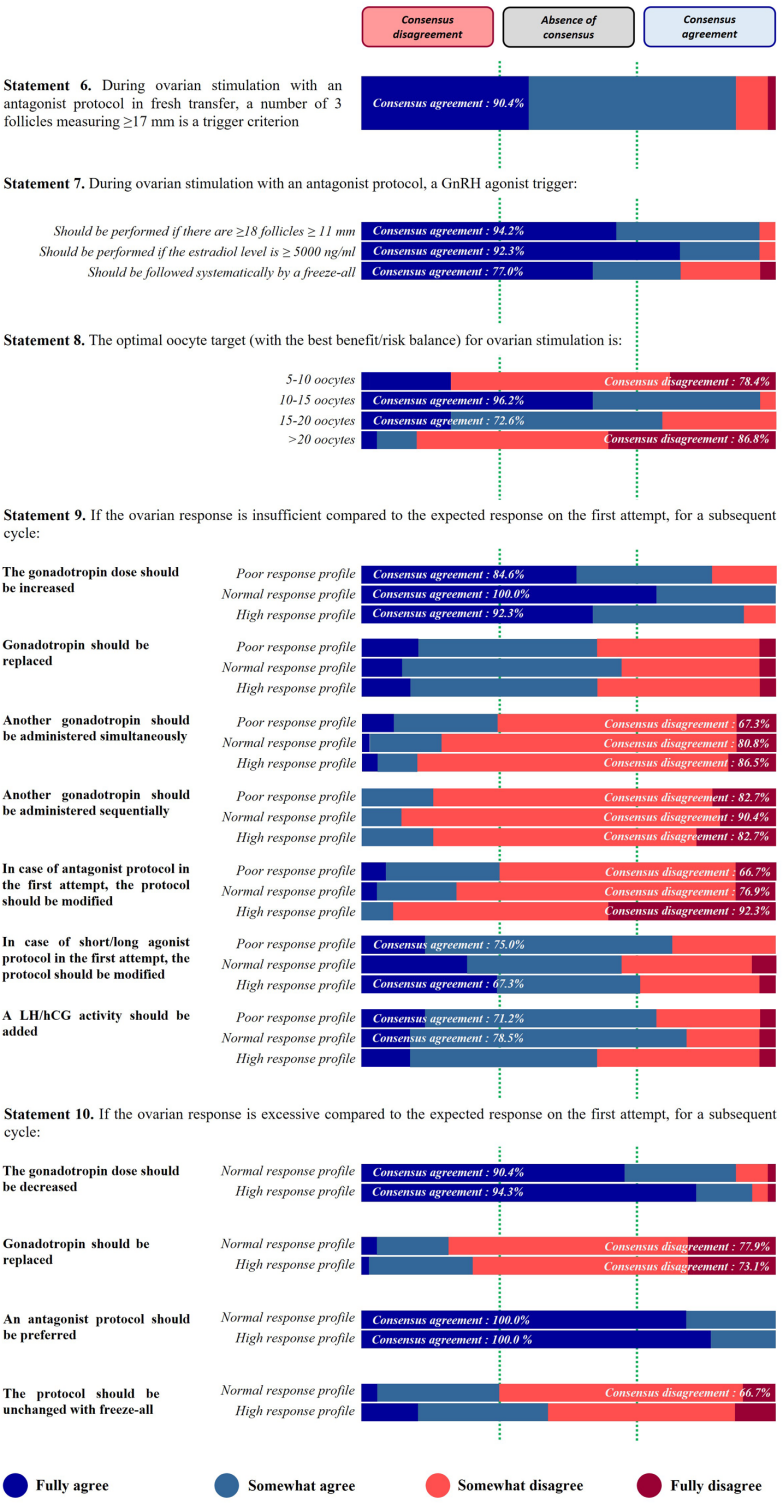
Ovarian response.

This statement received 90.4% of agreement from the panel of experts (Figure 3).

*Statement 7. During ovarian stimulation with an antagonist protocol, a GnRH agonist trigger should be…*

Among the three criteria proposed in this statement, two obtained a very strong consensus agreement (exceeding 90% of agreement) and the last one reached the consensus at a more limited level: used if there are ≥18 follicles ≥11 mm (94.2%); performed if estradiol level is ≥5,000 ng/ml (92.3%); and systematically followed by a freeze-all (77.0%) (Figure 3).

*Statement 8. The optimal oocyte target (with the best benefit/risk balance) for ovarian stimulation is…*

For this statement a consensus agreement of 96.2% was reached from the first round of voting for the target of 10–15 oocytes whilst a disagreement consensus of 86.8% was reached for the proposal >20 oocytes. After the second round of voting consensus agreement was also obtained for the proposal of 15–20 oocytes (72.6%) and disagreement consensus for the proposal of 5–10 oocytes (78.4%) (Figure 3).

*Statement 9. If the ovarian response is insufficient compared to the expected response on the first attempt, for a subsequent cycle…*

For the three profiles of patients, the panel of experts suggested to increase the gonadotropin dose for a subsequent cycle, with a strong level of consensus (84.6%, 100%, and 92.3% of agreement for low, normal, high response profiles, respectively) (Figure 3).

The panel of respondents did not advise to administer another gonadotropin whether simultaneously or sequentially, regardless of the response profile. No consensus was reached, for the replacement of gonadotropin in case of insufficient ovarian response. In addition, respondents did not recommend the modification of the protocol, in case of a GnRH antagonist protocol in the first attempt. The level of consensus progressively increased with the patient profile (66.7%, 76.9%, and 92.3% of disagreement for low, normal, and high responders, respectively). The experts agreed to modify the protocol in case of agonist protocol in the first attempt, but only in low (75.0% of agreement) and high response (67.3% of agreement) profiles (Figure 3).

Finally, adding LH/hCG activity was validated by the respondents for low and normal response profiles in case of insufficient ovarian response on the first attempt (71.2% and 78.5% of agreement, respectively) (Figure 3).

*Statement 10. If the ovarian response is excessive compared to the expected response on the first attempt, for a subsequent cycle…*

Logically, this statement has been designed for normo- and hyper-responsive profiles only. With a strong level of consensus agreement, the experts agreed to decrease the gonadotropin dose for a subsequent cycle in both profiles (>90% of agreement) (Figure 3).

The proposition “an antagonist protocol should be preferred” received the maximal level of agreement (100%) from the panel of experts for both normal and high responders (Figure 3). In contrast, respondents disagreed to replace the gonadotropin. A consensus disagreement was reached for both profiles (77.9% and 73.1% of disagreement for normal and hyper responders, respectively) (Figure 3).

Concerning the implementation of a freeze-all approach without modification of the protocol stimulation, no consensus was reached for the high response profile whereas consensus disagreement was achieved for the normal response profile. However, the consensus threshold was barely exceeded (66.7%) (Figure 3).

### 3.6 LH/hCG activity

*Statement 11. Supplementation of LH/hCG activity should be performed under the following conditions…*

**Figure 4:**
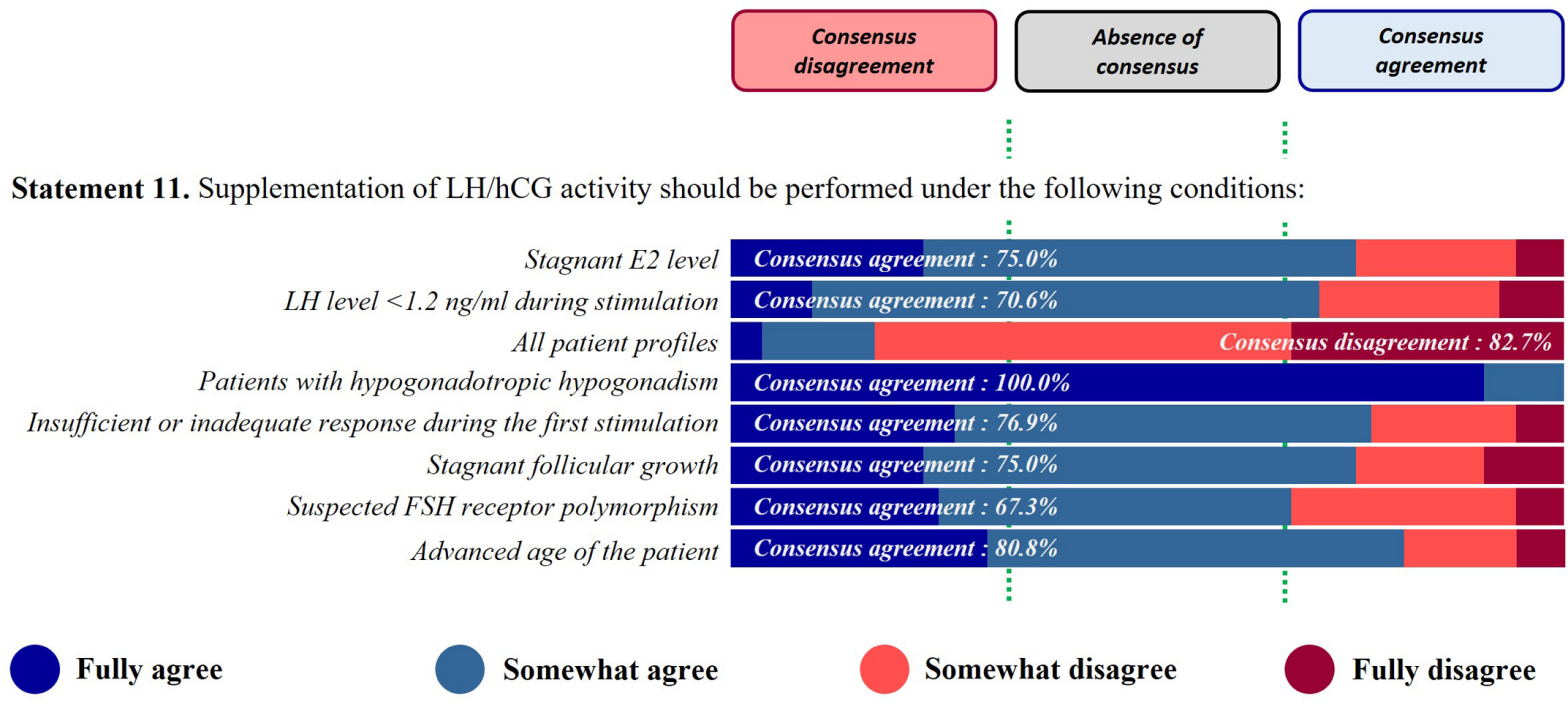
LH/hCG activity.

A consensus agreement was reached for the following suggestions: patient with hypogonadotropic hypogonadism (100%), advanced age of the patient (80.8%), insufficient or inadequate response during first stimulation (76.9%), stagnant E2 level (75.0%), stagnant follicular growth (75.0%), LH level <1.2 ng/ml during stimulation (70.6%) and suspicion of FSH receptor polymorphism (67.3%). The panel disagreed to prescribe a LH/hCG activity to all patient profiles (82.7% of disagreement) (Figure 4).

## 4 Discussion

This AMPLITUDE consensus brings new insights in clinical practices for the ovarian stimulation management. This study involved a large panel of fertility specialists practicing in France and Belgium. Despite the two rounds of voting, some propositions did not reach a consensus (agreement or disagreement). However, according to the literature, the optimal number of rounds seems to be 2 or 3, as a larger number of rounds will induce participant fatigue, questioning the relevance of obtained responses (10). Following this, a two-round Delphi consensus was conducted in this study. We can notice that several statements reached a consensus with a strong level of agreement or disagreement, improving the reliability of the results.

Firstly, this work strongly suggests that the initial dose of gonadotropins administered should be personalized for each patient profile. According to the literature, a daily dosage of 150 IU is considered as the standard dose. However, a subset of patients will have a low or a high response with this dosage. Thus, the objective is to obtain an optimal response, in order to improve the live birth rate and reduce the risk of hyperstimulation (11). Yet, the personalization of the initial gonadotropin dose implies a dose-response relationship between ovarian response and FSH dose. Despite limited data, the literature recommends personalizing the starting dose according to the patient profile (225–300 IU, 150–225 IU, and 100–150 IU in low, normal, and high responders, respectively) (11).

In this Delphi consensus, the panel of experts suggested that other parameters can be used concomitantly with AFC and AMH level for personalizing the gonadotropin dose: age, weight, BMI, nature of the cycle (ovulatory, dysovulatory, anovulatory), and the decision to adopt a fresh transfer or a freeze-all strategy, in all patient profiles. However, the experts do not recommend taking into account the patient history (gynecological and obstetrical histories reaching a disagreement consensus when no consensus being reached for general history, endometriosis, and parental project).

In the literature, none of the ovarian reserve tests is completely comprehensive in terms of sensitivity or precision but AFC and AMH are in the vast majority of cases good predictors of ovarian response (7, 8). There was a high agreement consensus for age as an important parameter for personalizing the gonadotropin dose. However, although age was also used to categorize responders’ profiles, especially the low responders (6, 12), age seems to be a better predictor of a successful pregnancy than of oocyte yield (7). Moreover, experts’ responses were aligned with the literature for weight and BMI that are classically described to influence fertility (13) and outcomes of infertility treatments (14, 15).

The absence of consensus can be explained for patients with endometriosis. Indeed, it is described that endometriosis alters ovarian function and can lead to a diminished ovarian reserve (16–18). Ovarian response after ovarian stimulation was also reported to be lower in women with ovarian endometriomas after adjusting for age, gonadotropin dose and AMH (17). However, as this impact depends on the localization of the lesions (ovarian lesions, especially), it can be assumed that some health professionals do not consider endometriosis when classifying their patients as hypo, normo-, or hyper-responders, as in some cases this pathology may have no effect on ovarian reserve.

In this Delphi we also questioned the relevance of a starting dose higher than 300 IU in low responders. Indeed, extremely high doses of gonadotropins have been used for decades in these patients. However, some studies described that FSH dosage beyond 300 IU do not improve ovarian response (19–21). Respondents agreed with this statement, suggesting that the maximum initial dose of gonadotropins for a low-responding patient should not exceed 300 IU. Several data in the literature support this result, including in first instance, the last ESHRE guidelines where a gonadotropin dose higher than 300 IU is not recommended for patients predicted as “poor responders” (8).

Respondents preferred a fresh transfer over freeze-all, when initiating ovarian stimulation in low and normal response patient profiles. Several studies evaluated the freeze-all benefits in patients undergoing IVF. The results showed that this strategy does not improve IVF outcomes in low responders (22). Data are controversial for normal responders: some authors described a positive effect on pregnancy rate in this population (22) when others concluded that freeze-all was not superior to fresh transfer for patients with normal ovarian response to stimulation (23). In high responders, data from the literature suggest beneficial effects of freeze-all, improving pregnancy rates (24).

Concerning the use of freeze-all when some events occur during stimulation, the panel of experts strongly recommended this option in case of high risk of OHSS, inadequate endometrium, new tubal or uterine cavity pathology, and premature elevation of progesterone. According to the literature, the freeze-all strategy was initially developed as a “rescue strategy” to counter the risk of OHSS in patients undergoing excessive ovarian response. Over the last decade, its indications were extended to other clinical conditions, i.e., endometrial, tubal, and uterine factors discovered during stimulation (thin endometrium, polyps, hydrosalpinx, endometritis, cervical anatomical features, cervical stenosis); slow-developing embryos or inadequate progesterone levels at the end of the follicular phase (however, various cut-offs have been reported, from 0.8 ng/ml to 1.8 ng/ml) (25–27).

This work then focused on the different trigger criteria, that appear to be consensual for the criterion of 3 follicles measuring ≥17 mm for a fresh transfer following an antagonist protocol according to the respondents. It is now well described that follicle size is linked to oocyte maturity. According to the literature, reaching the optimal follicular size on the day of oocyte retrieval would most likely yield a mature oocyte. However, although this optimal size range varies from one study to another: [12–19 mm]; [16–23 mm]; [23–28 mm] (28, 29), the 17 mm threshold seems to be the most commonly used criteria in studies and in daily practice, especially for antagonist protocols (29, 30).

Moreover, in the context of antagonist protocols, the use of GnRH agonist trigger was the preferred option if there are ≥18 follicles ≥11 mm as a predictor of severe OHSS or if the estradiol level is ≥5,000 ng/ml and this procedure should be systematically followed by a freeze-all according to respondents. These results agree with the literature where it is described that GnRH agonist trigger and a subsequent freeze-all strategy, when ≥18 follicles and E2 >5,000 ng/ml on the day of trigger, prevent the risk of OHSS (31–34).

According to the panel, the optimal oocyte target was 10–15 oocytes retrieved, at the first round of voting. However, another oocyte target reached a consensus at the second round: 15–20 oocytes. A possible explanation for this discordance may be due to the fact that the optimal oocyte target would be in the middle (around 15 oocytes) or to the lack of specification about the finality of the cycle, i.e., fresh or frozen transfer. Moreover, only two proposals that failed to reach consensus (5–10 and 15–20 oocytes) were put to the vote in the second round. It is therefore likely that respondents were encouraged to vote on these two proposals, in the absence of the more consensual proposal (10–15 oocytes).

In a Swedish study including 39,387 women undergoing an IVF procedure, the median number of oocytes retrieved was nine, with 0.5% OHSS. Authors also described that (1) live birth rate increased up to 11 oocytes retrieved and then levelled off and (2) the rate of severe OHSS increased with the number of oocytes retrieved, reaching 1% for 18 oocytes retrieved. These results suggest a shift in the balance between efficacy of treatment and patient safety regarding severe OHSS at approximately 18–20 oocytes (35–38). However, this refers to the live birth rate in a fresh transfer, while the cumulative live birth rate could be considered more relevant (39) both in a patient's and cost effectiveness perspective.

In some cases, an unexpected excessive ovarian response can occur. For the subsequent attempt, experts recommend decreasing the gonadotropin dose and to favor an antagonist protocol. Data from the literature are consistent with this result, as gonadotropin dose reduction is among the different strategies which should be considered for women with an unexpected high response (36). As well, antagonist protocols reduce the risk of severe OHSS by approximately 50%, compared to agonist protocols (34, 40).

In the context of hyper response, the necessity of performing a freeze-all approach without modification of the protocol is not consensual among the panel of respondents. Limited benefits of changing protocol after a first attempt have been described in the literature (41). Nevertheless, although freeze-all is the gold standard strategy to reduce OHSS for patients at risk of late OHSS, it does not completely prevent early-onset OHSS (42). Thus, a freeze-all strategy is not a fit to all strategy and a personalized approach should be preferred, based on patient characteristics, risk factors, and patient preference (43). For PCOS patients at high risk of OHSS, IVM could be considered as an alternative, but it is practiced in only a limited number of centers in France and Belgium (44).

Concerning patients with an unexpected suboptimal response, the consensus data suggest increasing the gonadotropin dose for the next treatment. According to the literature, different strategies can be set up in case of unexpected low ovarian response, including a higher FSH starting dose (37, 45, 46). The panel also agreed to modify the protocol in case of agonist protocol in the first attempt, but only in low and high response profiles. These results are in line with data from the literature, where a GnRH antagonist regimen is recommended in these populations (46, 47). The replacement of the gonadotropin does not seem to be an alternative to an inadequate response, but a LH/hCG supplementation can be suggested (48). Respondents are in favor of this strategy for unexpected insufficient ovarian response, but only in low and normal responders. In the literature, the benefits of LH supplementation are indeed more studied in low responders (45). The AMPLITUDE panel supported that unexpected hormonal levels (stagnant E2 or LH <1.2 ng/ml), hypogonadotropic hypogonadism, stagnant follicular growth, advanced age and suspected FSH receptor polymorphism, are additional indications for LH/hCG activity supplementation. This is consistent with literature findings. Indeed, in a systematic review of the literature, Hill et al., described that exogenous LH/hCG improved live birth rate. According to the authors, low responders and older patients (>35 years) are more likely to benefit from LH/hCG supplementation (47). Clinical evidence also largely supports LH/hCG supplementation in several patient types, including hypogonadotropic hypogonadism, patients older than 35 years old, low responders and unexpected low ovarian response, and profound suppression of LH serum concentrations (45, 47, 48).

FSH receptor (FSHR) polymorphisms can affect the level of FSH receptor expression in granulosa cells, that may lead to higher levels of FSH, lower ovarian sensitivity to FSH, and thus low ovarian response (49–51). In the literature, a systematic review suggested that LH supplementation in women presenting an impaired FSHR sensitivity to gonadotropins, may overcome the ovarian resistance (52).

It was important in this Delphi consensus to include the opinion of health professionals from different territories with their own particular socio-economical background. Sub-analyses by country were carried out but revealed no significant differences between the responses of healthcare professionals from France and those from Belgium. This Delphi was conceived and designed by and for Belgian and French health professionals, and as such it was not distributed on a wider scale to experts from other countries. This may constitute a non-negligible limitation of this study. However, there are no constraints with cost issues in the two countries, which could favor a higher diversity of applied treatment strategies to eventually identify the more clinically relevant protocol for different patients’ profiles.

We acknowledge that a consensus on the statements was not consistently achieved. This is a limitation off the study that may diminish the robustness of the decision-making process.

During the redaction phase, a particular attention was paid to ensure compliance with the guidelines for the reporting of consensus methods. Thus, the manuscript was drafted, reviewed, and adapted in accordance with the ACCORD guidelines (53).

In conclusion, data from this AMPLITUDE consensus support the idea of an optimized treatment for patients undergoing ART. In a context of lacking consensual data in the literature, this Delphi consensus provides interesting results, which hopefully will guide fertility specialists in their daily practice.

## Data Availability

The original contributions presented in the study are included in the article/Supplementary Material, further inquiries can be directed to the corresponding author.
