# Supplementary material for: From patient classification to optimized treatment in ART: the AMPLITUDE Delphi consensus
Source: Front Reprod Health. 2024 Sep 27;6:1467322. doi: 10.3389/frph.2024.1467322 (PMC11466932; doi:10.3389/frph.2024.1467322)
Supplement: Supplementary file 1 [file Table1.docx]

Supplementary Material

# Supplementary Tables

**Supplementary Table 1 – Panel of 52 experts involved in the AMPLITUDE consensus**

| Name | IVF Center | Round 1 | Round 2 |
| --- | --- | --- | --- |
| Florence ALEXANDRE | Hôpital Privé de La Chataigneraie, Clermont-Ferrand, Auvergne-Rhône-Alpes, France | X | X |
| Aurélie AMAR | Centre Sainte-Colette, Marseille, Provence-Alpes-Côte d’Azur, France | X | X |
| Mikaël AGOPIANTZ | Centre Hospitalier Régional Universitaire, Nancy, Grand-Est, France | X | X |
| Candice AUTIN | Centre Hospitalier Universitaire Saint-Pierre, Brussels, Belgium | X | X |
| Catherine AVRIL | Clinique Mathilde, Rouen, Normandie, France | X | X |
| Paul BARRIERE | Centre Hospitalier Universitaire, Nantes, Pays de la Loire, France | X | X |
| Aurélie BERDIN | Centre Hospitalier Régional Universitaire, Besançon, Bourgogne-Franche-Comté, France | X | X |
| Maud BIDET | Cabinet de Gynécologie Malakoff, Rennes, Bretagne, France | X | X |
| Myriam BOURRAT | Hôpital Privé Drôme Ardèche, Guilherand-Granges, Auvergne-Rhône-Alpes, France | X | X |
| Mathilde BOURDON | Hôpital Cochin, Paris, Ile-de-France, France | X | X |
| Véronique CHABERT-ORSINI | Cabinet Carré Saint-Giniez, Marseille, Provence-Alpes-Côte d’Azur, France | X | X |
| Martine CHOMIER | Institut Rhônalpin, Ecully, Auvergne-Rhône-Alpes, France | X | X |
| Rosalie CABRY | Centre Hospitalier Universitaire, Amiens, Hauts-de-France, France | X | X |
| Peter DE LOECKER | GZA Ziekenhuizen, Antwerpen, Vlaanderen, Belgium | X | X |
| Michel DE VOS | Brussels IVF, Universitair Ziekenhuis Brussel, Brussels, Belgium | X | X |
| Anne DELBAERE | Hôpital Erasme-Université Libre de Bruxelles, Brussels, Belgium | X | X |
| Annick DELVIGNE | Clinique CHC MontLégia, Liège, Wallonie, Belgium | X | X |
| Elodie DESCAT | Polyclinique Jean Villar, Bordeaux, Nouvelle-Aquitaine, France | X | X |
| Nathalie DHONT | Genk Institute for Fertility Technology, Genk, Vlaanderen, Belgium | X | X |
| Sophie DUBOURDIEU | Santé Atlantique, Nantes, Pays de la Loire, France | X | X |
| Sylvie EPELBOIN | Hôpital Bichat Claude Bernard, Paris, Ile-de-France, France | X | X |
| Imène FATFOUTA | Centre Matisse, Nice, Provence-Alpes-Côte d’Azur, France | X | X |
| André FORCE | Clinique du Val de l’Ouest, Ecully, Auvergne-Rhône-Alpes, France | X | X |
| Olivia GERVERAU | Centre Hospitalier Universitaire, Tours, Centre-Val de Loire, France | X | X |
| Anne GRELAT | Hôpital Privé Drôme Ardèche, Guilherand-Granges, Auvergne-Rhône-Alpes, France | X | X |
| Solenne GRICOURT | Hôpital Bichat Claude Bernard, Paris, Ile-de-France, France | X | X |
| Marie-Victoire GRIZEAU-SERGEANT | Centre Procrealis, La-Roche-sur-Yon, Pays de la Loire, France | X |  |
| Michaël GRYNBERG | Hôpital Antoine Beclère, Clamart, Ile-de-France, France | X | X |
| Veronika GRZEGORCZYK-MARTIN | Clinique Mathilde, Rouen, Normandie, France | X | X |
| Laurie HENRY | Centre Hospitalier Universitaire, Liège, Wallonie, Belgium | X | X |
| Claude HOCKE | Groupe Hospitalier Pellegrin, Bordeaux, Nouvelle-Aquitaine, France | X | X |
| Romain IMBERT | Chirec Hôpital Delta, Brussels, Belgium | X | X |
| Laëtitia JACQUESSON | Cabinet medical, Paris, Ile-de-France, France | X | X |
| Gwenola KERMONES | Groupe Hospitalier Diaconesses Croix Saint-Simon, Paris, Ile-de-France, France | X | X |
| Elisabeth LAMARCA | Cabinet de Gynécologie Altkirsch, Mulhouse, Grand-Est, France | X | X |
| Frédéric LAMAZOU | Clinique Ambroise Paré Hartmann, Neuilly-sur-Seine, Ile-de-France, France | X | X |
| Pascale LAURENT | Cliniques Universitaires Saint-Luc, Brussels, Belgium | X | X |
| Xenia LECHAT | Groupe Hospitalier Diaconesses Croix Saint-Simon, Paris, Ile-de-France, France | X | X |
| Chloé MAIGNIEN | Hôpital Cochin, Paris, Ile-de-France, France | X | X |
| Anne-Laure MARGULIES | Groupe Hospitalier Privé Ambroise Paré – Hartmann, Paris, Ile-de-France, France | X | X |
| Emmanuelle MATHIEU-D’ARGENT | Hôpital Tenon, Paris, Ile-de-France, France | X | X |
| Pascale MIRAKIAN | Hôpital Privé Natécia, Lyon, Auvergne-Rhône-Alpes, France | X | X |
| Pierre OGER | Cabinet médical Fertilité et Enfance, Rueil-Malmaison, Ile-de-France, France | X | X |
| Maud PASQUIER | Centre Hospitalier Intercommunal, Créteil, Ile-de-France, France | X | X |
| Maeliss PEIGNE | Hôpital Jean Verdier, Bondy, Ile-de-France, France | X | X |
| Noémie RANISLAVJEVIC | Centre Hospitalier Universitaire, Montpellier, Occitanie, France | X | X |
| Anne-Céline REYSS | Maison d'Asclepios , Avignon, Provence-Alpes-Côte d’Azur, France | X | X |
| Geoffroy ROBIN | Hôpital Jeanne de Flandre, Lille, Hauts-de-France, France | X | X |
| Marc SCHNEIDER | Clinique Belledonne, Saint-Martin d’Hères, Auvergne-Rhône-Alpes, France | X | X |
| Thérèse SCHWEITZER | Hôpital de Mercy, Peltre, Grand-Est, France | X | X |
| Dominic STOOP | Ghent University Hospital, Ghent, Vlaanderen, Belgium | X | X |
| Herman TOURNAYE | Brussels IVF, Universitair Ziekenhuis Brussel, Brussels, Belgium | X | X |

**Supplementary Table 2 – Statements approved by the AMPLITUDE Scientific Committee**

| Statements |
| --- |
| *Patient profile and personalization of the initial gonadotropin dose* |
| 1. The initial dose of gonadotropins administered should be personalized for each patient profile: poor, normal or high response profile |
| 2. In addition to the criteria defining the response profile (AFC and AMH), the parameters for personalizing the gonadotropin dose in a patient who is naïve to all stimulation treatments are as follow:  2.1 Poor response profile : Basal FSH level; Basal E2/FSH couple; Extreme values of LH (low or high); Age; Weight; BMI; Gynecological history (pelvic surgery); Obstetrical history (history of uterine rupture, ectopic pregnancy,…); Parental project (desired number of children); General history (chronic inflammation disease, cancer, etc…); Endometriosis (depending on the stage and the phenotype); Nature of cycle (ovulatory, dysovulatory, anovulatory); Opting for a fresh transfer or freeze-all  2.2 Normal response profile : Basal FSH level; Basal E2/FSH couple; Extreme values of LH (low or high); Age; Weight; BMI; Gynecological history (pelvic surgery); Obstetrical history (history of uterine rupture, ectopic pregnancy,…); Parental project (desired number of children); General history (chronic inflammation disease, cancer, etc…); Endometriosis (depending on the stage and the phenotype); Nature of cycle (ovulatory, dysovulatory, anovulatory); Opting for a fresh transfer or freeze-all  2.3 High response profile : Basal FSH level; Basal E2/FSH couple; Extreme values of LH (low or high); Age; Weight; BMI; Gynecological history (pelvic surgery); Obstetrical history (history of uterine rupture, ectopic pregnancy,…); Parental project (desired number of children); General history (chronic inflammation disease, cancer, etc…); Endometriosis (depending on the stage and the phenotype); Nature of cycle (ovulatory, dysovulatory, anovulatory); Opting for a fresh transfer or freeze-all |
| 3. The maximum initial dose of gonadotropins for a poor-responding patient should not exceed 300 IU for the first attempt |
| *Fresh transfer vs. Freeze-all* |
| 4. When initiating ovarian stimulation, a fresh transfer strategy is preferred as the first-line treatment for: poor response profile; normal response profile; high response profile |
| 5. Certain events discovered at initiation or during ovarian stimulation may lead to freezing all embryos. A freeze-all strategy is therefore preferable if the following events occur: High risk of hyperstimulation; Inadequate endometrium; New pathology of the uterine cavity; Premature elevation of progesterone (> 1.5 ng/ml); New diagnosis of tubal pathology (hydrosalpinx) |
| *Ovarian response* |
| 6. During ovarian stimulation with an antagonist protocol in fresh transfer, a number of 3 follicles measuring ≥17 mm is a trigger criterion |
| 7. During ovarian stimulation with an antagonist protocol, a GnRH agonist trigger: Should be performed if there are ≥18 follicles ≥11 mm; Should be performed if the estradiol level is ≥5000 ng/ml; Should be followed systematically by a freeze-all |
| 8. The optimal oocyte target (with the best benefit/risk balance) for ovarian stimulation is: 5-10 oocytes; 10-15 oocytes; 15-20 oocytes, >20 oocytes |
| 9. If the ovarian response is insufficient compared to the expected response on the first attempt, for a subsequent cycle:  9.1 Poor response profile: The gonadotropin dose should be increased; Gonadotropin should be replaced; Another gonadotropin should be administered simultaneously; Another gonadotropin should be administered sequentially; In case of antagonist protocol in the first attempt, the protocol should be modified; In case of short/long agonist protocol in the first attempt, the protocol should be modified; An LH/hCG activity should be added  9.2 Normal response profile: The gonadotropin dose should be increased; Gonadotropin should be replaced; Another gonadotropin should be administered simultaneously; Another gonadotropin should be administered sequentially; In case of antagonist protocol in the first attempt, the protocol should be modified; In case of short/long agonist protocol in the first attempt, the protocol should be modified; An LH/hCG activity should be added  9.3 High response profile: The gonadotropin dose should be increased; Gonadotropin should be replaced; Another gonadotropin should be administered simultaneously; Another gonadotropin should be administered sequentially; In case of antagonist protocol in the first attempt, the protocol should be modified; In case of short/long agonist protocol in the first attempt, the protocol should be modified; A LH activity should be added |
| 10. If the ovarian response is excessive compared to the expected response on the first attempt, for a subsequent cycle:  10.1 Normal response profile: The gonadotropin dose should be reduced; Gonadotropin should be replaced; An antagonist protocol should be preferred; The protocol should be unchanged with freeze-all  10.2 High response profile: The gonadotropin dose should be reduced; Gonadotropin should be replaced; An antagonist protocol should be preferred; The protocol should be unchanged with freeze-all |
| *LH/hCG activity* |
| 11. Supplementation of LH/hCG activity should be performed under the following conditions: Advanced age of the patient; Suspected FSH receptor polymorphism; Stagnant follicular growth; Insufficient or inadequate response during the first stimulation; Patients with hypogonadotropic hypogonadism; All patients profiles; LH level <1.2 ng/ml during stimulation; Stagnant E2 level |
